# Predictors of long time survival after lung cancer surgery: A retrospective cohort study

**DOI:** 10.1186/1471-2466-8-22

**Published:** 2008-10-27

**Authors:** Kjetil Roth, Tom Ivar Lund Nilsen, Elisabeth Hatlen, Karina Søvik Sørensen, Torstein Hole, Rune Haaverstad

**Affiliations:** 1 Dept. of Thoracic Medicine, Haukeland University Hospital, Bergen, Norway; 2 Dept. of Internal Medicine, Aalesund Hospital, Aalesund, Norway; 3 Section for Thoracic Medicine, Institute of Internal Medicine, University of Bergen, Bergen, Norway; 4 The Medical Faculty, Norwegian University of Science and Technology (NTNU), Norway; 5 Dept. of Cardiothoracic Surgery, Haukeland University Hospital, Bergen, Norway; 6 Institute of Surgical Sciences, The medical Faculty, University of Bergen, Bergen, Norway

## Abstract

**Background:**

There have been few reports regarding long time survival after lung cancer surgery. The influence of age and pulmonary function on long time survival is still not fully discovered. Some reports suggest that hospitals with a high surgical volume have better results. The aim of this study was to evaluate lung cancer surgery performed in a county hospital in terms of 30 days mortality, complications and predictors of long time survival.

**Methods:**

All patients operated with non-small cell lung cancer in the period 1993–2006 were reviewed, and 148 patients were included in the study. 30 days mortality and complications were analyzed by univariate analysis. Kaplan Meier plots were performed to display some of the univariate variables. Cox regression analysis was performed to find Hazard Ratios (HR) that predicted long time survival in univariate and multivariate analysis.

**Results:**

The overall 30 days mortality rate was 2.7%, whereas 36.3% had one or more complications after surgery. The median survival time was 3.4 years. In multivariate Cox regression analysis advanced preoperative stage predicted reduced long time survival with HR (95%CI) 1.63 (0.92, 2.89) and 4.16 (1.92, 9.05) for patients in stage IB and II-IV respectively, when compared to patients in stage IA. Age ≥ 70 years and FEV_1_<80% predicted reduced long time survival with HR (95%CI) 2.23 (1.41, 3.54) and 1.93 (1.14, 3.28) respectively, compared to age<70 years and FEV_1 _≥ 80%.

**Conclusion:**

Thirty days mortality and complication rate showed that lung cancer surgery can be performed safely in a county hospital with experienced thoracic surgeons. Early preoperative stage, age below 70 years and normal pulmonary function predicted long time survival.

## Background

The epidemiology of lung cancer has changed dramatically within the last 40 years. The incidence has increased, especially among women, and there are more adenocarcinomas. The number of surgical candidates in stage I and II disease have increased both for patients below and above 70 years [1-3]. There is an ongoing debate regarding centralisation of lung cancer surgery. Generally, hospitals with low volumes of lung cancer surgery have lower five years survival and more complications than teaching hospitals and hospitals with high volumes [4]. Predictors of higher survival rate or lower complication rate have been female gender [5-10], lower age [5,6,8,9,11,12], early preoperative stage [5,13], lobectomy [5,9,12-15], adenocarcinoma [8,13,16], no previous coronary heart disease [17-19], and normal pulmonary function tests [6,9,11,17,20,21]. Some of these predictors have been analysed only in univariate tests. There are as far as we know no previous reports from county hospitals where lung cancer surgery is performed with staff from larger teaching hospitals.

Aalesund Hospital in Norway is a county hospital with a catchment area of approximately 100 000 inhabitants. The aim of this study was to evaluate lung cancer surgery performed in a county hospital in terms of 30 days mortality, complications and long time survival, and to evaluate predictors of long time survival.

## Methods

A total of 149 operations in 148 patients at Aalesund Hospital from 1993 to 2006 with non-small cell lung cancer (NCLC) were retrospectively reviewed. One patient had two operations for different lung cancers and only the last operation was included in further analysis. The medical records of two patients were not found and these were excluded from the analysis of complications. The medical records of all patients were followed to 15.09.2007. Mortality data were available for all patients, but details about relapse were missing for four patients. All complications that appeared within 60 days after surgery were registered. Pulmonary embolism and deep venous thrombosis were included when they appeared within 90 days.

After the locally based specialist in thoracic surgery left in 1997, lung cancer surgery was taken over by three specialists from teaching hospitals in Tromsö and Trondheim. Adjuvant cytostatic treatment was introduced in November 2004. Only six patients received adjuvant chemotherapy, 10 patients had adjuvant irradiation therapy. Some advanced stage cases or high risk patients were initially referred to the regional hospital for treatment during the study period, but this was a minority. Pneumectomy was performed when there was a large central tumor or tumor invasion of a main bronchus, and the patients had a good pulmonary function with expected postoperative FEV_1 _above 1 litre.

The statistical analysis was performed in SPSS using Chi square tests and Hazard ratio from Cox regression for univariate analysis, and Hazard ratio from Cox regression for multivariate analysis of survival. Variables described as significant in previous studies were included in univariate analyses. All variables in the univariate analyses were included in the multivariate analysis. Kaplan Meier plots were performed to estimate median survival time and to visualize some of the univariate variables.

The Regional Medical Ethics Committee and the Norwegian Social Science Data Service approved the study.

## Results

The baseline characteristics of the patients are displayed in table 1. A malignant diagnosis was obtained for 111 (75%) of the patients before surgery. In 43 cases (29.1%) the diagnosis was obtained after inconclusive bronchoscopy by a percutaneous approach. The median time from bronchoscopy to diagnosis was five days (n = 111, range 1–71 days) and the median time from diagnosis to surgery was 17 days (n = 111, range 0–127). Adenocarcinoma was the most prevalent histology. The mean age was 67.3 years (95% CI: 47.0, 87.6). Of the 16 patients operated with preoperative stage II-IV, distribution was stage IIA: 2, IIB: 3, IIIA: 6, IIIB: 3 and IV: 2. The patients in stage IIIB had more than one lesion in the affected lobe. In stage IV one patient had intrapulmonal metastasis and one patient had cerebral metastasis. The postoperative pathological stage was I, II, III, and IV for 89, 38, 15, and six patients respectively.

**Table 1:** Baseline characteristics of 148 patients operated for lung cancer

	n	%
***Sex***		

Female	48	32.4

Male	100	67.6

***Age***		

< 70 years	86	58.1

≥ 70 years	62	41.9

***Preoperative stage***		

IA (T1 N0 M0)	64	43.2

IB (T2 N0 M0)	68	45.9

II-IV	16	10.8

***Operation type***		

Lobectomy	97	65.5

Pneumonectomy	30	20.3

Bilobectomy	16	10.8

Wedge	5	3.4

**Histology**		

Adenocarcinoma	73	49.3

Squamous cell carcinoma	53	35.8

Large cell carcinoma	14	9.5

Other non-small cell carcinoma	8	5.4

***Coronary heart disease***		

No previous coronary disease	107	72.3

Previous coronary disease	41	27.7

**Pulmonary function**		

FEV1 ≥ 80%	62	41.9

FEV1<80%	77	52.0

Indeterminate	9	6.1

Data are presented as number of positive samples/all samples (%).

The overall complication rate was 36.3%. Pneumonia (16.4%) and respiratory failure (6.8%) were the most common complications (Table 2). Table 3 describes the univariate analyses of complications, 30 day survival and 1 year survival. The overall 30 day mortality rate was 2.7% (four patients) and one year mortality rate was 18.9%. Age above 70 years predicted a higher rate of complications (p < 0.002), higher 30 days mortality (p = 0.02) and higher one year mortality (p < 0.001). Pneumonectomy and coronary heart disease were predictors of higher 30 day mortality in univariate analysis; and actually no early mortality was seen after lung resections less than pneumonectomy. Causes of early death were respiratory failure (n = 3) and multiorgan failure (n = 1). Six patients were re-operated because of complications. Of the 144 patients eligible for follow-up, 47.9% had relapse of lung cancer. Median time to relapse was 44.9 months. The relapse rate was 69.7% in the group with FEV_1_<80% compared to 30.3% in the group with FEV_1 _≥ 80% (n = 138, p = 0.01).

**Table 2:** Hospital morbidity (n = 146)

	N	%
Any complications	53	36.3

Wound infection	5	3.4

Postoperative bleeding	3	2.1

Pneumonia	24	16.4

Sepsis	1	0.7

Bronchopleural fistula	2	1.4

Empyema	1	0.7

Drainage of fluid or air	5	3.4

Deep venous thrombosis	1	0.7

Pulmonary embolism	3	2.1

Respiratory failure	10	6.8

Myocardial infarction	5	3.4

Heart failure	3	2.1

Atrial fibrillation	9	6.2

Renal failure (creatinine>140)	2	1.4

Multi organ failure	1	0.7

Cerebral infarction	2	1.4

Other complications	4	2.7

Data are presented as number of positive samples/all samples (%). Two patients were excluded because of insufficient information.

**Table 3:** Univariate analysis of 30 days mortality, complications and 1 year mortality.

	n	30 days mortality	p	Complications*	p	1 year mortality	p
***Sex***			0.16		0.37		0.07

Female	48	0%		31.3%		10.4%	

Male	100	4%		38.8%		23.0%	

***Age***			0.02		0.002		<0.001

< 70 years	86	0%		25.9%		9.3%	

≥ 70 years	62	6.5%		50.8%		32.3%	

***Preoperative stage***			0.09		0.18		0.04

IA (T1 N0 M0)	64	0%		31.3%		10.9%	

IB (T2 N0 M0)	68	5.9%		36.4%		22.1%	

II-IV	16	0%		56.3%		37.5%	

***Operation type***			0.001		0.29		0.11

Lobectomy	97	0%		31.3%		15.5%	

Pneumonectomy	30	13.3%		46.7%		33.3%	

Bilobectomy	16	0%		40%		18.8%	

Wedge	5	0%		60%		0%	

**Histology**			0.57		0.35		0.21

Adenocarcinoma	73	1.4%		33.3%		13.7%	

Squamous cell carcinoma	53	3.8%		42.3%		22.6%	

Large cell carcinoma	14	7.1%		42.9%		35.7%	

Other non-small cell carcinoma	8	0%		12.5%		12.5%	

***Coronary heart disease***			0.03		0.84		0.91

No previous coronary disease	107	0.9%		36.8%		18.7%	

Previous coronary disease	41	7.3%		35.0%		19.5%	

**Pulmonary function**			0.22		0.40		0.86

FEV1 ≥ 80%	62	4.8%		33.9%		19.4%	

FEV1<80%	77	1.3%		40.8%		18.2%	

Statistical analysis: χ2.

*Two patients excluded because of insufficient information.

**Figure 1:**
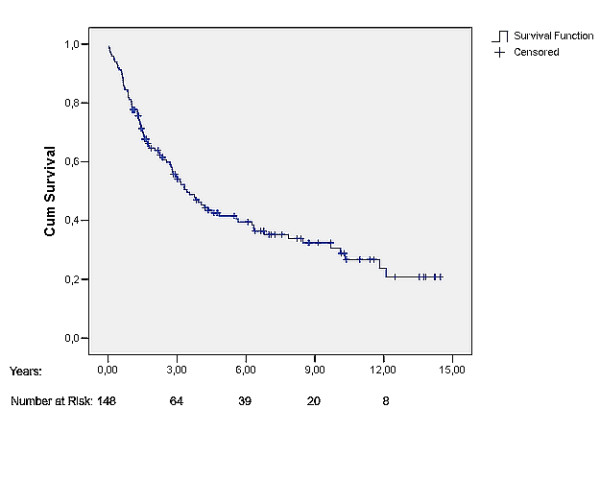
Postoperative survival curve (Kaplan-Meier plot) for all patients.

**Figure 2:**
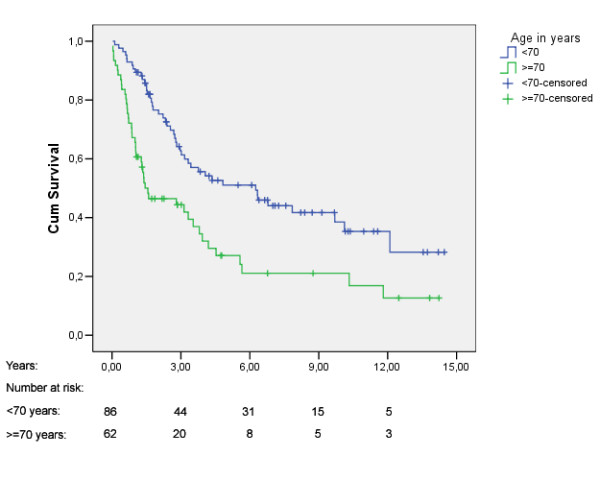
Kaplan-Meier plot for overall survival stratified by age.

**Figure 3:**
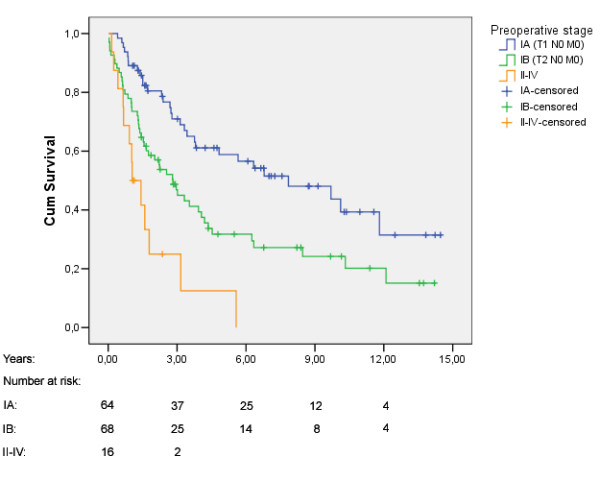
Kaplan-Meier plot for overall survival stratified by preoperative stage.

The median survival time was 3.4 years (95% CI: 2.4, 4.5). Five years overall survival rate was 41.6% (Fig 1). Figure 2, 3, 4 shows Kaplan Meier plots for long time survival according to age, preoperative stage, and pulmonary function. Cox regression analysis showed a significant relation between long time survival and age, type of operation, preoperative stage and pulmonary function in univariate analysis. There was no significant relation between long time survival and gender or previous coronary heart disease. Age, preoperative stage and pulmonary function remained significant in the multivariat analysis (Table 4).

**Figure 4:**
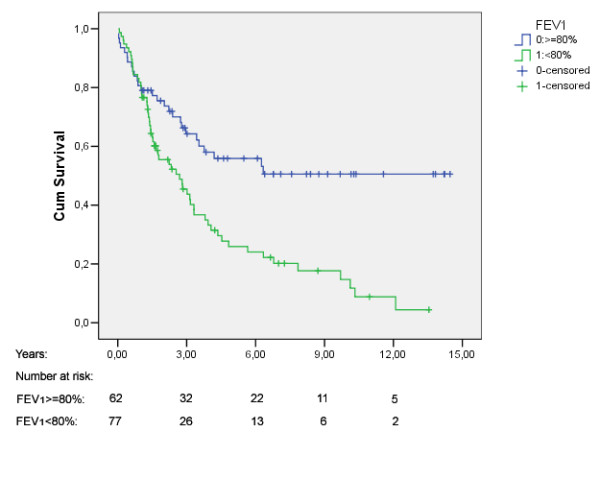
Kaplan-Meier plot for overall survival stratified by preoperative pulmonary function.

**Table 4:** Hazard ratio (HR) for survival

	Univariate analysis			Multivariate analysis		
	HR	95% CI	p	HR	95% CI	p

***Sex***						0.945

Female	1			1		

Male	1,28	0.82, 2.0	0.28	1.02	0.59, 1.75	

***Age***						0.001

< 70 years	1			1		

≥ 70 years	2.04	1.35, 3.09	0.001	2.23	1.41, 3.54	

***Preoperative stage***						0.003

IA (T1 N0 M0)	1			1		

IB (T2 N0 M0)	2.00	1.27, 3.17	0.003	1.63	0.92, 2.89	

II-IV	4.59	2.34, 9.02	<0.001	4.16	1.92, 9.05	

***Operation type***						

Lobectomy	1			1		0.57

Pneumectomy	2.14	1.34, 3.43	0.002	1.37	0.78, 2.43	

Bilobectomy	0.94	0.48, 1.85	0.85	0.84	0.40, 1.76	

Wedge	0.70	0.17, 2.88	0.62	0.70	0.16, 3.15	

**Histology**						0.32

Adenocarcinoma	1			1		

Squamous cell carcinoma	1.51	0.96, 2.37	0.08	0.86	0.48, 1.54	

Large cell carcinoma	2.60	1.36, 4.98	0.004	1.79	0.80, 3.99	

Other non-small cell carcinoma	0.63	0.19, 2.04	0.44	0.80	0.24, 2.67	

***Coronary heart disease***						0.73

No previous coronary disease	1			1		

Previous coronary disease	1.27	0.81, 1.99	0.31	0.91	0.54, 1.54	

**Pulmonary function**						0.013

FEV1 ≥ 80%	1			1		

FEV1<80%	2.30	1.45, 3.65	<0.001	1.93	1.14, 3.28	

HR = Hazard Ratio. Statistical analysis: Cox Regression analysis method = enter.

p value: univariate: Wald, multivariate: Likelihood ratio.

## Discussion

The 30 days mortality rate of 2.7% in this study was comparable or lower than previously reported from high volume hospitals for lung cancer surgery [4,12]. Five years overall survival rate of 41.6% is comparable to previous reports from larger centres [5,8,13]. The higher 30 days mortality and morbidity rate among elderly patients and following pneumonectomy have previously been described [7,11,12], but the relation have not been significant in all studies [19]. The early complication rate is prone to information bias in a retrospective study, but our report of 36.3% with ≥ 1 complication was within the same range as in previous reports [4,7,12,13].

Age above 70 years and a more advanced preoperative stage were predictors of reduced long time survival in this study as well as in some previous studies [5,13,22]. Thus, these variables should routinely be included in future multivariate analyses. The increased survival of female gender has previously been described [5,10], but like comparable studies, we could not find any significant effect of gender [13].

FEV_1 _< 80% predicted reduced long time survival by multivariate analysis. The predictors of long time survival have been described in different studies. FEV_1 _can be treated linearly, with cut off points, or in terms of FEV_1_/FVC ratio. One previous study applied FEV1<70% of predicted and found only a small influence on long time survival [13]. The present study found a marked difference between patients with FEV_1 _≥ 80% versus FEV_1_<80% of predicted value. The cause of death was not registered in all patients, but the relapse rate in patients with FEV_1 _<80% was higher than in the group with FEV_1 _≥ 80%. We did not have sufficient information about smoking to adjust for pack-years. In patients registered for their smoking habits, the consumption of tobacco was similar in the two groups. The explanation for a higher mortality rate in the group with FEV_1_<80% is not known and should be further investigated. A hypothesis may be that it is more likely to develop minor pre-cancerous lesions when tobacco smoke has caused lung injury in patients suffering from chronic obstructive lung disease.

Strengths of this study were the long observation time and that all operated patients were included. Thus, the study reflected the complete patient group and the hard end points were easily assessed, except for the missing early postoperative data in two patients. A major limitation is the retrospective design of the study. Although all operated patients were included, there may have been selection bias as the surgeons selected their cases. Referral of some advanced and high risk cases to the regional hospital may also have contributed to a better long time survival in this patient group. The main variables well known to influence on survival were included, but the relatively low number of patients in this study made it impossible to adjust for too many factors in the multivariate analysis.

## Conclusion

This study has shown that lung cancer surgery performed in a county hospital with surgeons from teaching hospitals can be performed with good results. The 30 days mortality rate was low and the complication rate was in the range of previous studies. Predictors of reduced long time survival were age over 70 years, advanced preoperative stage and FEV_1_<80%. The effect of pulmonary function on long time survival should be investigated in a larger study, the quality of surgery should be registered prospectively.

## Competing interests

The authors declare that they have no competing interests.

## Authors' contributions

Two of the authors (EH and KSS) reviewed the patients journals. RH, EH and KSS staged the disease based on preoperative information. RH, TH and KR planned and performed the investigation, KR and TILN performed the statistical analyses.

## Pre-publication history

The pre-publication history for this paper can be accessed here:


